# MRI for an Acute Secondary Site Complication in Post-arthroplasty Management: Narrative Review of Safety Concerns for an Implanted Hip and Knee Joint

**DOI:** 10.7759/cureus.22319

**Published:** 2022-02-17

**Authors:** Lavindra Tomar, Gaurav Govil, Pawan Dhawan

**Affiliations:** 1 Department of Orthopedics, Max Super Speciality Hospital, Patparganj, Delhi, IND

**Keywords:** surgical staples., replacement, secondary site, complication, orthopaedic implant, knee arthroplasty, hip arthroplasty, safety guidelines, implant migration, magnetic resonance imaging

## Abstract

Arthroplasty in the elderly may present with acute or late-onset complications unrelated to an implanted arthroplasty joint. Magnetic resonance imaging (MRI) evaluation of an acute onset complication in the immediate post-arthroplasty scenario presents safety concerns. An arthroplasty surgeon's dilemma relates to the loosening, heating, or migration of implanted hip or knee joints.

We present a representational case scenario for discussion. A hip arthroplasty patient presenting with hemiplegia in the immediate postoperative period necessitated an MRI evaluation for the brain with an additional angiogram. A knee arthroplasty patient presenting with lower limb weakness in the immediate postoperative period necessitated an MRI evaluation of the brain. Loosening of surgical metallic clips used for wound closure and the instability or loosening of recently implanted hip and knee joints pose significant safety concerns for the arthroplasty surgeon. The confirmatory diagnosis of the secondary site complication in the acute post-arthroplasty perioperative period, however, allowed the allied super-specialist to plan the management protocol.

A review of the literature suggests that the use of nonferromagnetic elements in implanted joints with the use of cement or the press-fit method of implantation during arthroplasty has high safety margins. The staples used for wound closure have significant strength to hold the wound without any disruption or dehiscence during the MRI imaging. The metallic artifacts associated with an implanted joint do not interfere in the evaluation of the secondary site MRI.

MRI can be safely done in a well-fixed joint of non-ferromagnetic elements. The review of literature also suggests that MRI can be done even in the presence of skin staples for the assessment of an acute secondary site complication in a post-arthroplasty patient. The risk-to-benefit ratio though needs to be applied for imaging a secondary site.

## Introduction and background

Arthroplasty has become a commonly performed procedure with a proven success rate to overcome painful hip and knee pathologies. It has helped especially the geriatric population to attain favorable outcomes with an improved quality of life [1-3]. The volume of total joint arthroplasty has increased with the increasing life expectancy of the elderly population [4]. In the elderly with coexistent medical comorbidities, as compared to the general population, there are increased chances of acute as well as late-onset post arthroplasty complications to occur [5-7].

Any early or late postoperative arthroplasty complication requires meticulous evaluation. Primary site complications related to arthroplasty occur more frequently in the late postoperative period [5-6]. A complication related to an implanted joint may require local site imaging by radiographic examination, specialized magnetic resonance imaging (MRI), computerized tomography, or radionucleotide scan evaluation for diagnostic confirmation [8-9]. A few uncommon complications presenting in the acute post-operative phase are stroke, paralysis, or fat embolism syndrome which may warrant an appropriate imaging modality for an early assessment and effective management [10-13]. Though unrelated to the site of the implanted joint, evaluation of an acute onset complication may require a secondary site MRI to diagnose and necessitate remedial measures by an allied super-specialist for the timely management plan. MRI evaluation of an acute onset complication in the immediate post arthroplasty scenario presents safety concerns for the implanted joint. An arthroplasty surgeon's dilemma relates to its loosening, heating, or migration of the implanted hip or knee joint.

We present a representational case study one each for a hip and knee arthroplasty which required secondary site MRI for the evaluation of an acute complication with due risk-benefit ratio stratification and informed consent of the patient. The diagnosis was confirmed and early measures were initiated for the management by an allied super-specialist. The possibility of the loosening or dislodgement of surgical metallic clips and arthroplasty implants in an acute post-operative phase posed significant safety concerns with a poor understanding and significant dilemma for the arthroplasty surgeon.

We reviewed the literature regarding the safety concerns for undertaking an MRI in an acute secondary site complication following arthroplasty of the hip and knee joint. The aim was to allay the fears, and review the literature to provide an evidence-based suggestion, for getting a secondary site MRI evaluation in the acute post arthroplasty phase by the arthroplasty surgeon.

## Review

Case scenarios

Case 1

**Figure 1:**
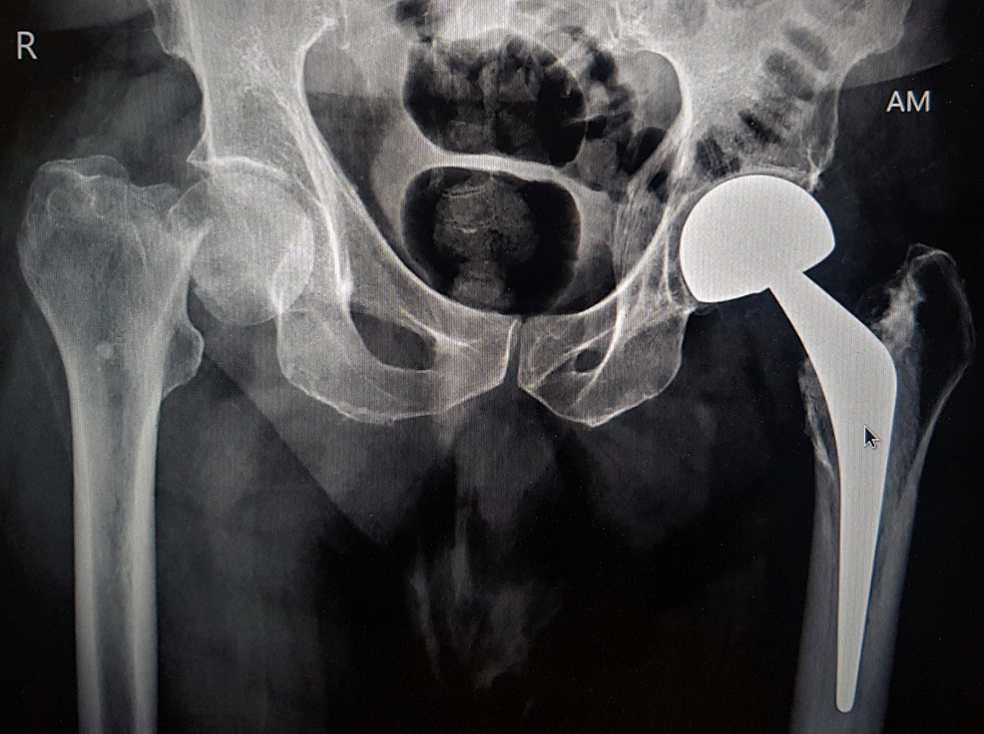
Pelvis radiograph with a right hip fracture of the neck femur and left hip bipolar prosthesis in situ

An elderly male presented with a slip and fall at home, with an injury to the right hip region. The injury was radiologically confirmed as a displaced fracture neck femur right hip (Figure 1). There was a past history of left hip cemented bipolar arthroplasty eight years ago.

**Figure 2:**
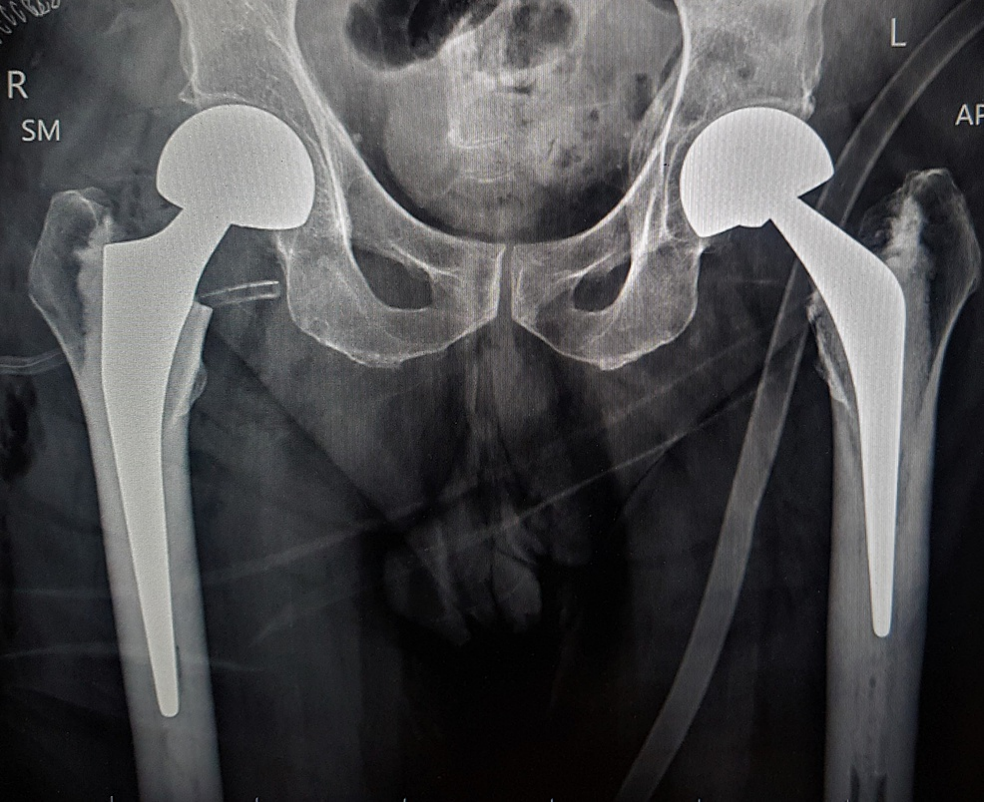
Pelvis radiograph with both hips' bipolar prosthesis in situ

There was a history of cerebrovascular episodes one year ago, which had recovered and allowed him to ambulate without any support. He underwent neurological assessment by a neurologist in the preoperative assessment. The carotid Doppler was essentially normal. The cardiac evaluation was done by dobutamine stress echocardiography, which was negative for inducible ischemia. Prophylactic thrombo-prophylaxis and the use of compression mechanical devices were initiated in the preoperative period for the prevention of deep vein thrombosis. Right hip cemented modular bipolar hemiarthroplasty was done under regional anesthesia on the third day of the injury (Figure 2).

In the immediate postoperative period, impaired movements of the right-sided upper and lower limbs were observed. The vital parameters were maintained, and he was conscious and responding to commands. A neurologist reviewed and suggested an MRI evaluation to assess for the intracranial pathology.

**Figure 3:**
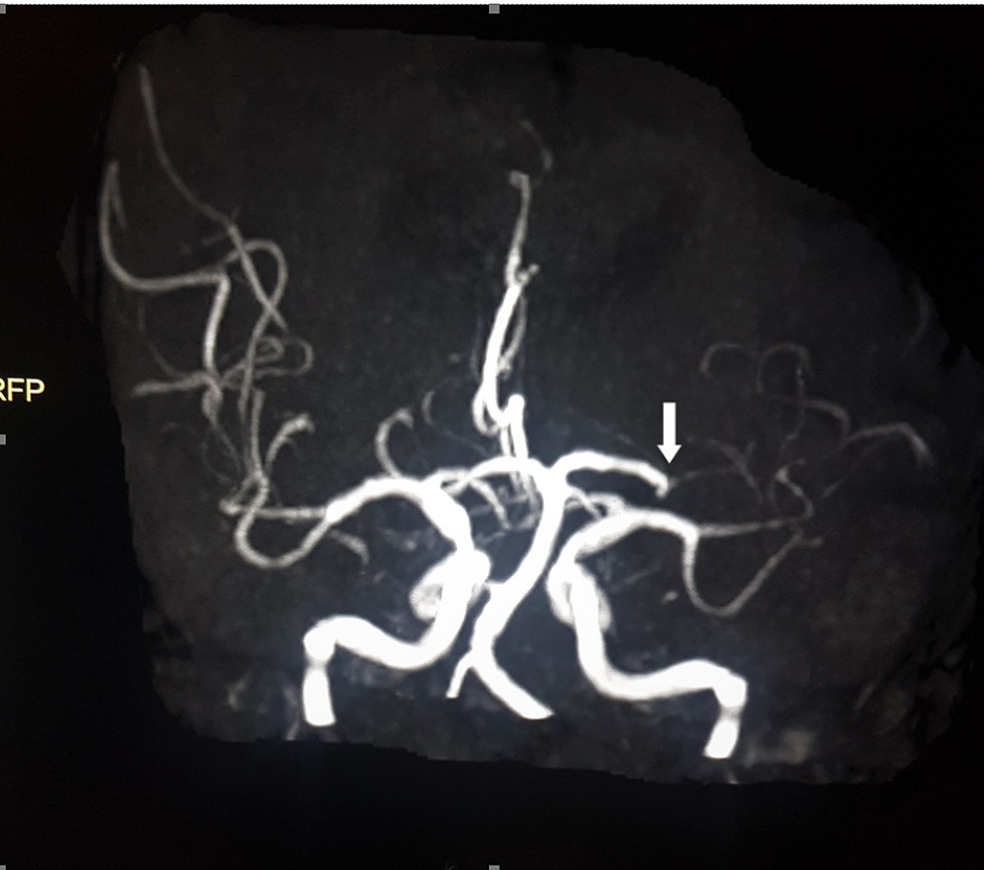
MRI angiogram of the circle of Willis showed occlusion of the left middle cerebral artery (white arrow)

The dilemma was how to proceed for MRI? We presumed that heating, dislodgement of skin staples, or loosening of the recently implanted hip would be a safety hazard for an MRI. A lead apron was placed along the pelvic area and operated wound site strapped. Post MRI, the local site was re-inspected to assess the displacement of sutures, abnormal wound protuberance, or abnormal skin change locally. Passive range of hip movements was assessed for any deformity, limb length discrepancy, or restriction. No local-site complications were observed. MRI brain revealed an acute left insular infarct. Angiography revealed occlusion with the absence of flow-related enhancement in the left middle cerebral artery territory (Figure 3).

A neurologist was consulted and management was initiated. A period of observation in an intensive care unit with assisted supervised physiotherapy for immediate management was initiated. Walker support mobilization was possible. He needed prolonged physiotherapy to regain independent mobilization status.

Case 2

**Figure 4:**
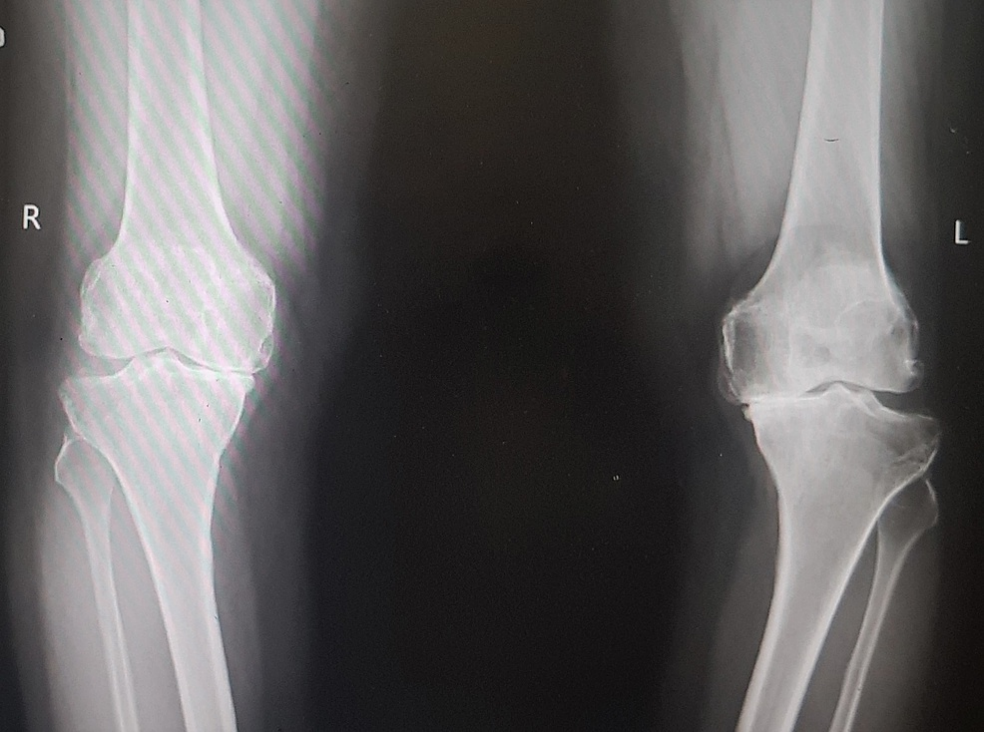
Bilateral knee radiographs (anteroposterior standing view) shows severe osteoarthritis changes

A 60-year-old female suffering from bilateral osteoarthritis of knees (Figure 4) with a history of associated type 2 diabetes underwent a planned simultaneous total knee arthroplasty under regional anesthesia with combined spinal-epidural medication.

**Figure 5:**
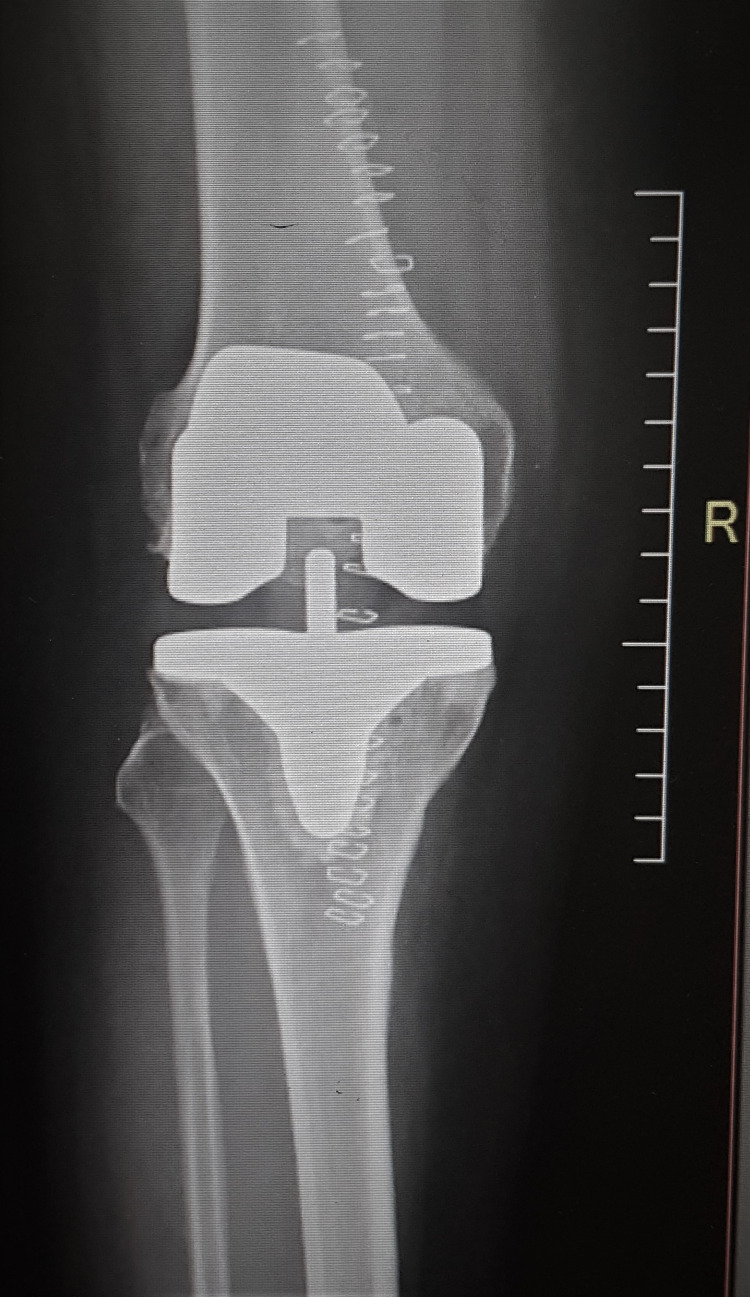
Radiograph of the right knee (anteroposterior view) with a stable implant in the immediate postoperative period

**Figure 6:**
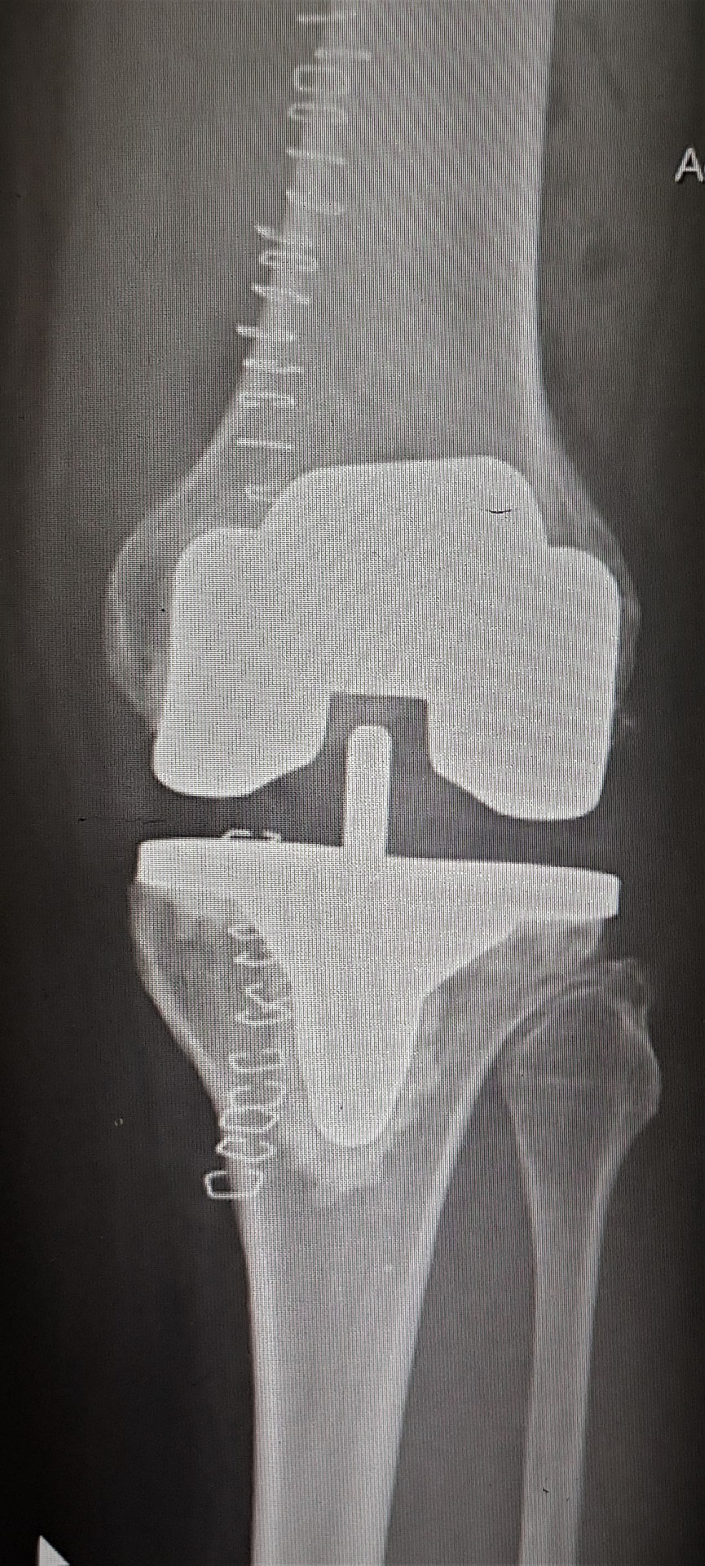
Radiograph of the left knee (anteroposterior view) with a stable implant in the immediate postoperative period

The immediate postoperative radiographs of the knee showed well-aligned joints (Figures 5-6).

**Figure 7:**
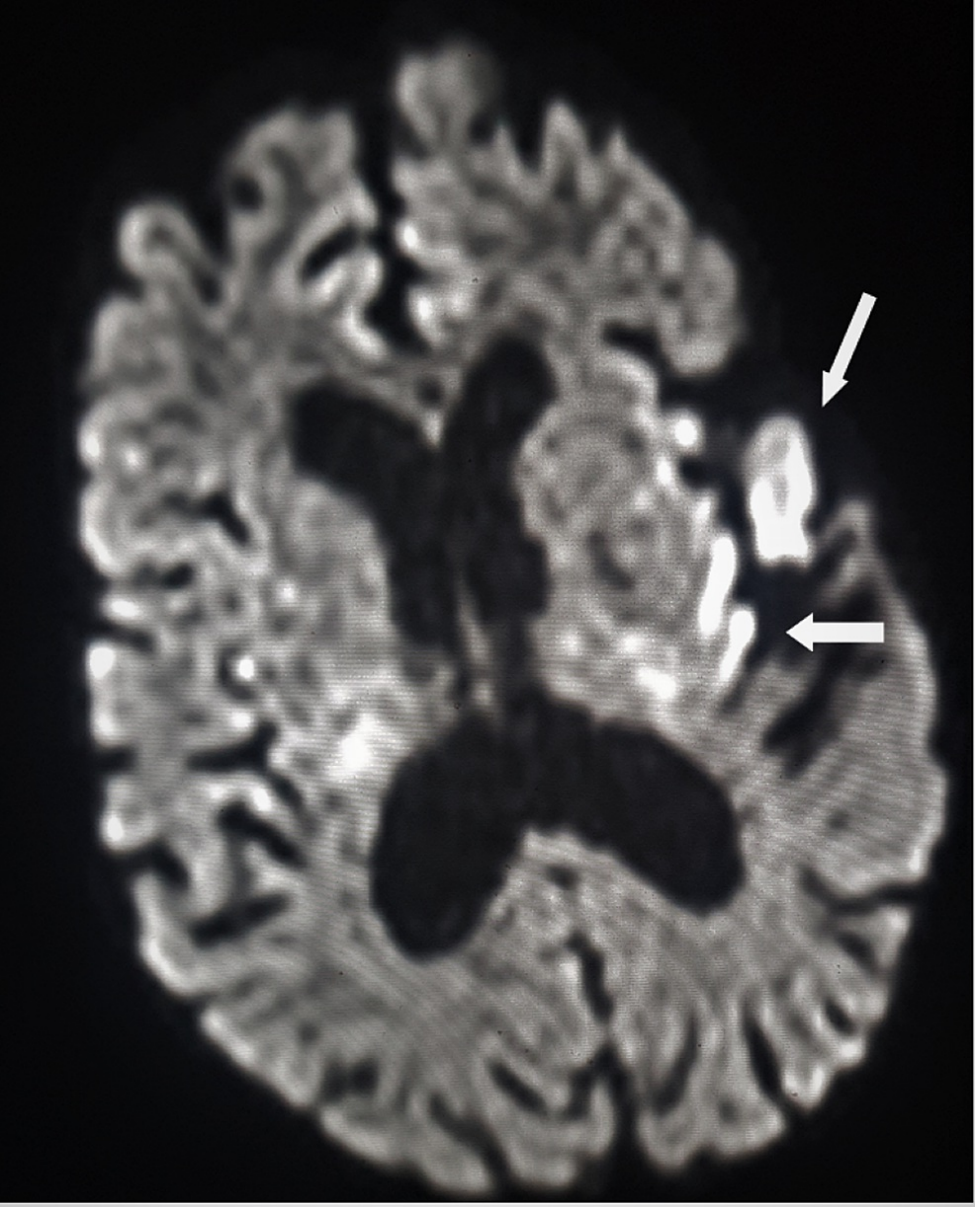
MRI brain with focal areas of hyperintense signal on DWI with signal drop seen in the left frontal periventricular region and left insular cortex, suggesting acute infarct (white arrow) DWI: diffusion-weighted imaging

In the immediate postoperative period on the second day of surgery, weakness of the right lower limb was noted. Discontinuation of epidural medication, however, showed no signs of improvement. The persisting weakness required close observation along with a neurological assessment by the neurologist. The neurologist required an MRI brain evaluation to identify and ascertain the relevant pathology. The presence of skin staples and recently implanted knees posed significant challenges for the management team. The risk-benefit ratio was evaluated based on the literature and safety guidelines from the manufacturer manual. Written informed consent from the patient was taken. An MRI imaging of the brain was done with a lead apron along the pelvic and knee area with the operated wound site strapped. Post MRI, an assessment and review of the local site for the displacement of sutures or abnormal protuberance or skin change locally was done. The passive range of movement of both knee joints was assessed to be within routine ranges of knee motion. No local-site complication was noted. MRI brain revealed an acute infarct of the left frontal periventricular region (Figure 7).

The neurologist initiated medical management for the infarct. A period of observation in an intensive care unit and assisted supervised physiotherapy for her immediate management followed. Subsequently, with prolonged physiotherapy, she regained her ambulatory status.

Discussion

MRI has been a valuable diagnostic tool in musculoskeletal imaging due to its lack of ionizing radiation and excellent soft-tissue contrast. However, its use in the acute postoperative period following orthopedic implantation has been a surgeon's dilemma [14-15]. The safety of MRI regarding implant migration and heating of soft tissues has been a debatable issue [4,14-15]. Image artifacts from in-vicinity metal require additional considerations in image interpretation [14]. A risk-to-benefit ratio should be evaluated when the need for MRI arises [14].

MRI in Problematic Primary Hip and Knee Arthroplasty Site

MRI imaging of hip and knee arthroplasty for assessment of the primary site complications related to the implanted joint was initially not favored due to image distortion and metal artifacts. The role of MRI with optimized conventional and advanced pulse sequences has allowed improved evaluation of periprosthetic arthroplasty-related complications [16]. It may guide the surgeons in planning surgical interventions in suspect and severe bone loss cases [17-19].

A review of 23 studies to evaluate the role of MRI as a diagnostic modality for the assessment of problematic knee arthroplasty emphasized that MRI was accurate and reliable for diagnosing infection, loosening and wear, and malalignment after knee arthroplasty, however, for other areas of concern related to instability, arthrofibrosis, or patellofemoral complaints, there are limited and inconclusive studies [20]. However, there was no mention regarding the time duration of conducting the test from the day of arthroplasty and safety concerns or complications, if any, related to implanted joint. However, most of them were conducted in the late post arthroplasty phase [20].

MRI Safety Concerns in the Immediate Post-arthroplasty Period

In the immediate post-arthroplasty period, any acute complication at a secondary site challenged the decision-making regarding the evaluation by a site-specific MRI. There are no specific guidelines and the surgeons may lack an understanding posing difficulties in the management in the post-operative period. The potential concerns are stapled dislodgement or loosening, image artifacts, heating of ferromagnetic implants, and possible soft tissue damage to the implanted joint.

MRI in the Presence of Surgical Staples 

Surgical staples are commonly used for the closure of orthopedic surgical wounds. It allows for early and effective closure of arthroplasty wounds. However, safety issues related to the dislodgement and heating of soft tissues locally during MRI imaging have been a concern [15].

Metal surgical clips have a wide range of uses, including wound closure, bowel anastomoses, and vascular hemostasis. When clips constructed from ferromagnetic metals are exposed to a magnetic field, movement or heating of clips can occur, causing local tissue damage [21]. A discussion between the surgeon and radiologist is recommended if clips are placed in friable tissues, as most manufacturers urge caution in this situation [21].

The study by Gill and Shellock [22] assessed the MRI issues at 3-Tesla for 61 metallic skin closure staples and vessel ligation clips. Results of the study showed that each surgical implant showed minor magnetic field interactions (20 and 27 degrees, which is acceptable from a safety consideration). They also concluded that heating was not substantial (highest temperature change = 1.6°C). They concluded that the artifacts may create issues if the area of interest is in the same area or close to the respective surgical implant [22].

A study conducted on a pig foot, which was incised and repaired with staples suggested that MRI scanning in the presence of stainless-steel surgical staples seems safe, and no heating effect was noticed; rather, a drop-in temperature was identified though with a possible explanation due to the standard cooling process of the MRI [15]. No dislodgement or wound dehiscence was noted with skin staples. A "heat sink" by cold compresses in the area of staples has been recommended [15].

A review based on 15 studies about orthopedic implant migration, torque, and radiofrequency-induced heating gave further directions for its safety [14]. The Shellock conclusion still holds regarding the safety of MRI at 1.5 T or less in the immediate postoperative period in patients with passive nonferromagnetic implants, but if an implant is weakly magnetic, surgeons should wait six to eight weeks after the procedure to allow tissue ingrowth and help prevent the implant from shifting [14-15,23].

MRI and Artifacts in Imaging

The movement of metallic implants has been a potential risk only when the object possesses significant magnetism [15]. Medical-grade stainless steel is composed of ferromagnetic elements such as iron, nickel, and chromium. However, they are balanced in such a way that the resulting alloy is nonmagnetic [15]. Titanium or oxidized zirconium, in comparison to cobalt-chromium, has reduced artifacts on imaging [24]. A femoral stem generated fewer artifacts as compared to the acetabular component or femoral head due to the parallel orientation of the stem to the magnetic field. There are larger artifacts with a spherical geometry of the acetabulum or femoral head [24].

MRI has become the most sensitive imaging method to evaluate painful hip arthroplasty at late stages to detect and quantify the extent of periprosthetic osteolysis and to detect wear-induced synovitis, leading to aseptic loosening and implant failure [24]. Fast spin-echo and short inversion recovery minimize image distortion [8,14].

The representational case description and the literature review should help alleviate the safety concerns for an arthroplasty surgeon in a recently implanted joint and guide them in decision-making to manage the hip or knee arthroplasty case.

## Conclusions

Advanced MRI imaging techniques and no clear evidence of MRI-induced loosening or implant migration allow a justified use of conducting an MRI. An arthroplasty surgeon can conduct the secondary site MRI imaging in a hip and knee post-arthroplasty for an acute postoperative complication.

The risk-to-benefit ratio needs to be applied for imaging a secondary site. It can be done safely with due risk stratification in a well-fixed joint of non-ferromagnetic elements in the presence of skin staples for an evaluation of acute secondary site pathology for effective post-arthroplasty management.
